# Supplementary material for: Evaluating the Phenotypic and Genomic Characterization of Some Egyptian Phages Infecting Shiga Toxin-Producing Escherichia coli O157:H7 for the Prospective Application in Food Bio-Preservation
Source: Biology (Basel). 2022 Aug 5;11(8):1180. doi: 10.3390/biology11081180 (PMC9404725; doi:10.3390/biology11081180)
Supplement: Supplementary file 1 [file biology-11-01180-s001.zip › biology-1801052-supplementary.pdf]

Supplementary Materials

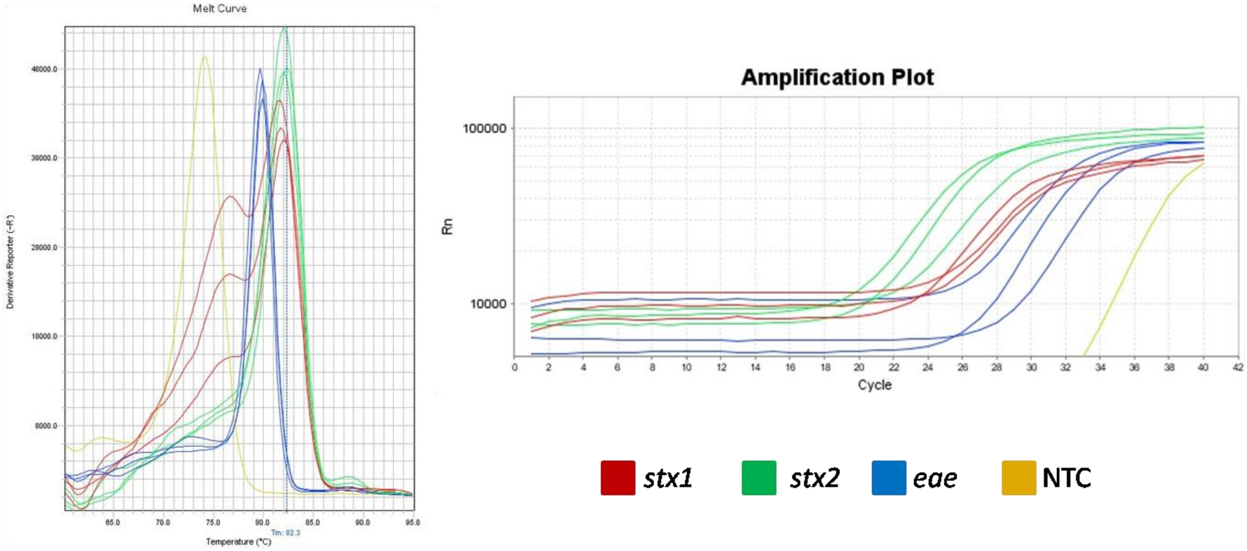

Figure S1. Quantitative real time PCR (RT-PCR) results. The amplification plot and melting curves of *stx1*, *stx2* and *eaeA* gene expression. The RT-PCR confirmed the presence and successful expression of the three tested genes in Shiga toxin-producing *E. coli* O157:H7 wild type strain 93111.

Table S1. Analysis of structural proteins of selected four phages.

Bands	Ladder		STEC P1		STEC P2		STEC P3		STEC P4	
	Band M. W.	Band density	Band M. W.	Band density	Band M. W.	Band density	Band M. W.	Band density	Band M. W.	Band density
1	170	40,338	119.22	30,457	120.63	31,146	122.39	28,673	142.51	30,279
2	130	40,530	109.69	28,263	106.51	30,677	106.16	29,054	126.28	31,884
3	100	44,272	104.75	28,393	98.75	27,505	100.16	29,191	121.34	34,633
4	70	39,537	97.33	26,650	92.39	30,870	90.98	34,237	107.92	33,990
5	55	43,729	88.51	32,704	84.63	28,687	79.33	28,671	100.16	31,356
6	40	37,560	75.1	25,376	74.04	29,967	72.98	31,661	91.69	33,480
7	35	40,951	69.09	32,328	63.8	27,313	66.27	31,211	81.1	33,656
8	25	39,633	62.03	29,406	58.51	27,255	53.21	37,674	75.45	33,593
9	15	39,455	48.62	36,924	50.39	38,420	36.27	27,794	69.09	40,836
10	10	41,798	26.38	24,381	28.15	25,269	29.56	26,648	63.09	32,507
11	-	-	15.44	23,731	22.15	23,486	19.68	25,493	55.33	41,303
12	-	-	7.68	20,662	13.68	27,939	14.74	25,089	46.5	30,746
13	-	-	1.32	22,179	7.68	24,406	9.44	26,305	37.68	32,384
14	-	-	-	-	1.32	25,214	-	-	30.97	34,618
15	-	-	-	-	-	-	-	-	15.09	34,611
16	-	-	-	-	-	-	-	-	6.62	34,040
17	-	-	-	-	-	-	-	-	NA	38,273
Total M. W.	480	-	825.16	-	822.63	-	801.17	-	1170.82	-

Table S2. The closest phages to *Escherichia* phage ST2 obtained from the NCBI GenBank.

S	Genome	Size	GC %	Query cover %	E value	Identity %	Accession No.	ANI	DDH
**1**	*Escherichia*-phage vB_EcoM_PhAPEC2	167318	37.7	96	0	98.92	KF562341.1	98.1	81.6
**2**	*Shigella*-phage Shf125875	169062	37.6	94	0	97.38	KM407600.1	96.58	67.4
**3**	*Escherichia*-phage SF	168695	37.6	95	0	97.46	MH359124.1	96.59	69.3
**4**	*Escherichia*-phage vB_EcoM_WFK	164590	37.6	90	0	97.61	MK373775.1	96.82	69.6
**5**	*Escherichia*-phage vB_EcoM_WFL6982	164279	37.6	90	0	97.6	MK373774.1	96.82	69.5
**6**	Enterobacteria-phage RB69	167560	37.7	94	0	97.47	AY303349.1	96.65	69.4
**7**	*Escherichia*-phage vB_EcoM_JS09	169148	37.6	92	0	96.55	KF582788.2	95.89	55.8
**8**	*Shigella*-phage phi25-307	167544	37.5	91	0	96.44	MG589383.1	95.74	56
**9**	Shigella-phage JK42	168306	37.6	91	0	96.33	MK962756.1	95.95	55.3

Table S3. The closest phages to *Escherichia* phage ST4 obtained from the NCBI GenBank.

S	Genome	Size	GC %	Query cover %	E value	Identity %	Accession No.	ANI	DDH
**1**	*Escherichia* phage vB_EcoP_PhAPEC7	71,778	43.3	86	0	95	KF562340.1	93.85	80.4
**2**	*Escherichia* phage vB_EcoP_PhAPEC5	71,248	43.5	83	0	95	KF192075.1	93.66	75.2
**3**	*Escherichia* phage vB_EcoP_G7C	72,917	43.4	83	0	95	HQ259105.1	93.16	77.3
	Escherichia phage PD38	72,213	42.9	84	0	93	MH669274.1	91.76	75.1
**4**	*Escherichia* phage phi G17	68,270	43.5	80	0	93	MH358458.1	92.61	73.5
**6**	Enterobacteria phage Bp4	72,583	42.9	84	0	93	KJ135004.2	91.8	75.4
**7**	ECBP1	69,855	42.7	81	0	93	JX415535.1	91.46	77.2
**8**	*Escherichia* phage PGN829.1	74,437	42.9	87	0	92	MH733496.1	91.86	77.8
**9**	*Enterobacter* phage IME11	72,570	43.1	81	0	92	JX880034.1	91.61	73.8
